# Faith Healing

**Published:** 1886-06

**Authors:** 


### SELECTIONS.

### Faith Healing.

New interest has been awakened in the question of miraculous healing, by recent faith-cure conventions in Chicago and other cities. A report of the convention in this city, made by the editor of the *Northwestern Advocate*, from personal observation, states that "two hundred rose when asked if any present had ever been miraculously healed in answer to prayer. Over thirty rose when asked if any had been cured of organic disease."

That there have been many remarkable cases of faith-healing is not disputed. In truth, there is very little healing without faith. Dependence upon physicians in cases of serious illness involves a sublime faith. Hardly less impressive is the faith of many who "have no faith in doctors," and depend upon "nature," or their own powers of recuperation, aided by a few simple remedies of their own prescribing. (Nothing has more power to restore the body to a healthy condition than faith on the part of the invalid, in God, in medical skill, and in his own ability to throw off disease.) A rational faith relies on and unites all these in cases of confirmed and dangerous illness. It is not wise, it is hardly moral, for any class of men, however strong their faith in God, to habitually ignore medical skill, though in many cases all that is necessary is to possess the patient with a strong faith in his own power to overcome his disability. Faith-healers profess to ignore all natural remedies, and do refuse medical advice; but they often use the very remedy a skillful physician would prescribe. They urge the invalid to assume that he is healed, and to exert himself accordingly, and thus inspire a confidence in his own ability to use the faculties God has given him, and this enables him to really do so.

The sickness of not a few of the persons supposed to have been miraculously healed, has been protracted, if not originally caused, by a lack of faith in their own powers and refusal to exert them. Many a poor man is bedridden to-day, who could walk, if he could only be made to believe it. A man may as well be a cripple as to be under the inexorable belief that he can not move his limbs. In an article on faith-healing in the last *Contemporary Review*, Dr. Moxon says:

"When people's own supply of faith is so deficient that they fail utterly to realize their power over their own cases, then it is that whoever or whatever can suddenly or gradually increase their faith, is able to restore their power. The law may be formulated thus: In so far as the disease is a lack of faith, in just that degree is the cure an act of faith-healing.

He gives, in illustration, the case of a boy sent to him for treatment, who, by a fall which injured his spine, had been "totally paralyzed in the legs for five years, and when he came to the hospital, he could not feel when his legs were touched or pinched, nor could he move them in the least degree." By dint of threatening, coaxing, and bribing, the doctor induced him to "walk, leaning upon and pushing a chair before him. In two weeks he was able to run races in the hospital park." If this boy had received the same treatment at Lourdes or Bethsan, in addition to the praying and anointing, and had been cured, he would have been proclaimed as a shining example of miraculous healing.

We need have no hesitancy in admitting that a multitude of genuine cases have been wrought by faith. The question is: "Are these cures miracles?" Modern science of the agnostic type says, No; for a miracle is impossible. Intelligent Christian faith is less dogmatic. It does not claim to know as much concerning what God can and can not do as does agnosticism. For aught we know, God can work as genuine miracles in these days as in the time of Christ; and we regard it as more reverent and modest to admit that he may do so on adequate occasions than to presume to set metes and bounds to his work. It is not a question of his power to work miracles, but one of fact as to whether modern faith-cures present all the necessary credentials of a miracle. That many of them resulted from the influence of spiritually quickening minds on diseased bodies, is doubtless true, and so far they are justly attributable to divine power; but as regeneration and sanctification by the Holy Spirit are not miracles in the commonly received sense of the term, so the restoration of the body to health, resulting from a divine work in the soul, is not a miraculous cure. The cures so wrought are cases of neurotic diseases in which the influence of the mind on the body is most powerful.

Great injury is done by these extravagant claims to the persons who make them, and to the cause of religion. Since the claims are made by devoutly religious people, in the name of religion, their evident fallacy leads irreligious people to regard spiritual conversion as a similar delusion, and doubt all religion. The effects upon the patients themselves is stated by Dr. Moxon as follows:

"The condemnation of the movement is cruel confusion of sickness with sin, and of healing with holiness. What more agonizing than the state of heart and soul and body when some poor wretch has gone to Bethshan, and striven in vain for recovery there, having been made to believe that Christ will prove the acceptance of the soul by the healing of the body? The poor creature leaves, down-hearted, thinking, 'My body is not cured. I am told that Christ heals the body as readily as he saves the soul. He has not healed my body. He will not save my soul. I am lost forever.' It is for this cause that what is called 'Faith-Healing' deserves condemnation as being cruel and heartless and injurious to the most suffering and pitiable of our fellow-men. The faith of the sick is not fair game for the sport of unhealthy religious enthusiasm. Faith must not be taught to fail the poor and those in deep trouble: and healing is everything to the bread-winner suddenly stricken with disabling disease. Sickness is too serious to be trifled with by fanatics. In this direction the faith-healing movement approaches criminality.—*The Advance.*

